# Clinical Characteristics, Management, and 30-Day Mortality Predictors in an 18-Year Pediatric Candidemia Cohort

**DOI:** 10.3390/jof12060445

**Published:** 2026-06-17

**Authors:** Coskun Ekemen, Ulgen Celtik, Ezgi Kiran Tasci, Kubra Cebeci, Melike Yasar Duman, Suleyman Emre Karauzum, Gizem Guner Ozenen, Gulcihan Ozek, Nihal Karadas, Eda Ataseven, Gulizar Turan, Miray Karakoyun, Ahmet Celik, Dilek Yesim Metin, Gulhadiye Avcu, Zumrut Sahbudak Bal

**Affiliations:** 1 Division of Infectious Diseases, Department of Pediatrics, Medical School of Ege University, 35100 Izmir, Turkey; coskunekemen@hotmail.com (C.E.); emrekarauzum@hotmail.com (S.E.K.); gul_akbas@yahoo.com.tr (G.A.); 2 Department of Pediatric Surgery, Medical School of Ege University, 35100 Izmir, Turkey; ulgenceltik1988@gmail.com (U.C.); celikahmet969@gmail.com (A.C.); 3 Division of Gastroenterology, Hepatology and Nutrition, Department of Pediatrics, Medical School of Ege University, 35100 Izmir, Turkey; ezgikiran@gmail.com (E.K.T.); miraykarakoyun@hotmail.com (M.K.); 4 Division of Intensive Care Unit, Department of Pediatrics, Medical School of Ege University, 35100 Izmir, Turkey; cebecikubramd@gmail.com (K.C.); dr.gulizarturan@gmail.com (G.T.); 5 Department of Medical Microbiology, Medical School of Ege University, 35100 Izmir, Turkey; melike.yasar@ege.edu.tr (M.Y.D.); dilekyesimb@yahoo.com (D.Y.M.); 6 Department of Pediatric Infectious Diseases, Dr. Behcet Uz Children’s Diseases and Surgery Training and Research Hospital, 35210 Izmir, Turkey; gzmguner86@gmail.com; 7 Division of Hematology and Oncology, Department of Pediatrics, Medical School of Ege University, 35100 Izmir, Turkey; gulcihanozek@yahoo.com (G.O.); drnihalozdemir@yahoo.com (N.K.); eda.ataseven@ege.edu.tr (E.A.)

**Keywords:** Candida, candidemia, children, catheter removal, mortality, antifungal resistance, source control

## Abstract

Pediatric candidemia is a major cause of invasive fungal infections in hospitalized children, but long-term data on epidemiology, management, and mortality predictors remain limited. We conducted an 18-year retrospective cohort study of 465 pediatric candidemia episodes at a tertiary referral center in western Turkey between 2008 and 2025. The primary outcome was crude 30-day mortality; associated factors were assessed using univariable analyses, Kaplan–Meier estimates, and multivariable logistic regression. Non-albicans *Candida* species predominated, with *Candida parapsilosis* as the most frequent isolate (46%). Central venous catheters were present in 88.4% of episodes. Crude 30-day mortality was 10.8%. Reduced survival was observed among patients without catheter removal and among those with thrombocytopenia, severe neutropenia, or immunosuppressive therapy. Among 341 episodes classified as central line-associated bloodstream infections, crude 30-day mortality differed significantly by catheter removal timing. Mortality was 4.8% with catheter removal within 72 h versus 13.1% without early removal (*p* = 0.022). Using a 48 h threshold, mortality was 3.1% with removal within 48 h versus 12.3% without removal within 48 h (*p* = 0.029). In multivariable analysis, failure to remove the catheter was the strongest independent factor associated with mortality (adjusted odds ratio, 6.63; 95% confidence interval, 2.85–15.42; *p* < 0.001). Antifungal resistance patterns were not consistently associated with mortality. In this large pediatric candidemia cohort, 30-day mortality was mainly associated with host vulnerability and modifiable management factors, underscoring the importance of timely source control.

## 1. Introduction

Pediatric candidemia remains a major cause of invasive fungal infections in hospitalized children and is consistently reported among the leading causes of nosocomial bloodstream infections worldwide [1,2,3]. Despite advances in diagnostics and antifungal therapy, candidemia continues to be associated with prolonged hospitalization, increased healthcare costs, and substantial mortality, with reported 30-day mortality rates ranging from approximately 10% to over 40% in pediatric cohorts [4,5,6,7,8].

Over the past two decades, the epidemiology of candidemia has shifted from *Candida albicans* toward non-albicans *Candida* (NAC) species. Although *C. albicans* remains clinically important, species such as *C. parapsilosis*, *C. glabrata*, and *C. tropicalis* now predominate in many pediatric centers [1,9,10,11]. In particular, *C. parapsilosis* has emerged as an important pediatric pathogen, likely related to its ability to form biofilms on intravascular devices and its association with catheter-related bloodstream infections. This changing epidemiology is further complicated by increasing antifungal resistance, particularly to fluconazole, although its impact on clinical outcomes remains debated.

The development of pediatric candidemia is multifactorial and strongly associated with central venous catheters, prolonged intensive care unit stay, exposure to broad-spectrum antibiotics, and total parenteral nutrition. High-risk populations include children with malignancies, intestinal failure, or those requiring intensive care support. Current management strategies emphasize early diagnosis, prompt initiation of appropriate antifungal therapy, and effective source control, including timely removal of central venous catheters, as catheter retention has been associated with adverse outcomes [11,12].

Identifying patients at highest risk of death is essential to optimize management strategies. Although previous studies have proposed predictors such as septic shock, thrombocytopenia, and immunosuppression, long-term cohort data integrating epidemiological trends, management practices, and independent predictors of 30-day mortality in pediatric populations remain limited.

In this 18-year retrospective cohort study including 465 episodes of pediatric candidemia at Ege University Children’s Hospital, a major tertiary referral center in western Turkey, we aimed to characterize clinical features, species distribution, and management practices, and to identify independent risk factors for crude 30-day mortality.

## 2. Materials and Methods

### 2.1. Study Design and Setting

This retrospective cohort study was conducted at Ege University Children’s Hospital, a tertiary-care referral center in western Turkey. The study period spanned from January 2008 to December 2025. Pediatric patients aged 28 days to 18 years with at least one episode of candidemia during hospitalization were screened for eligibility.

### 2.2. Study Population

Candidemia was defined as the isolation of *Candida* species from at least one blood culture obtained from a peripheral vein or an intravascular catheter in the presence of compatible clinical findings. To avoid multiple counting of episodes within the same hospitalization, only the first candidemia episode per hospitalization was included. Episodes with incomplete microbiological, clinical, or outcome data were excluded.

Persistent candidemia was defined as continued *Candida*-positive blood cultures for ≥72 h after initiation of systemic antifungal therapy. Breakthrough candidemia was defined as infection developing after ≥3 days of systemic antifungal prophylaxis or treatment. *Candida* colonization was defined as isolation of the same species from non-sterile sites within 14 days before or after candidemia.

Neutropenia was defined as an absolute neutrophil count (ANC) <1.5 × 10^3^/µL and severe neutropenia as ANC <0.5 × 10^3^/µL. Thrombocytopenia was defined as a platelet count <150 × 10^3^/µL.

Mortality outcomes were assessed at 14 and 30 days following candidemia diagnosis. The primary study outcome was 30-day crude all-cause mortality, defined as death from any cause within 30 days of candidemia diagnosis. In critically ill children with malignancy, intensive care exposure, or multiple comorbidities, distinguishing deaths directly attributable to candidemia from those related to underlying disease is often challenging. Therefore, consistent with prior pediatric invasive candidiasis cohorts that have used overall 30-day mortality as an objective endpoint [13], we selected 30-day crude all-cause mortality as the primary analytic outcome.

Fourteen-day candidemia-attributable mortality was defined as death occurring within 14 days of the first positive blood culture for *Candida* species in the absence of an alternative identifiable non-infectious or microbiologically documented cause of death. Attribution was determined through detailed retrospective review of electronic medical records by the study investigators. In cases of uncertainty, deaths were conservatively classified as non-attributable.

For subgroup analyses, central venous catheter presence and central line-associated bloodstream infection (CLABSI) classification were evaluated separately. Episodes were classified as CLABSI according to the Centers for Disease Control and Prevention (CDC) National Healthcare Safety Network (NHSN) criteria [14]. Briefly, CLABSI was defined as a laboratory-confirmed bloodstream infection due to *Candida* species occurring in a patient with an eligible central venous catheter (CVC) in place on the date of event (DOE) or the day before, in the absence of an alternative primary source of bloodstream infection. Therefore, the presence of a CVC alone was not considered sufficient for CLABSI classification. During retrospective classification, the source of the positive blood culture was also reviewed. Catheterized episodes with *Candida* growth only in peripheral blood cultures, without concomitant catheter-drawn blood culture positivity, were not included in the CLABSI subgroup used for catheter-removal timing analyses. NHSN laboratory-confirmed bloodstream infection (LCBI) criteria were retrospectively applied through detailed reviews of electronic medical records. Episodes meeting NHSN criteria for secondary bloodstream infection were excluded from the CLABSI subgroup. Analyses of early catheter removal were restricted to episodes classified as CLABSI.

### 2.3. Data Collection

Demographic, clinical, and laboratory data were retrospectively extracted from electronic medical records using a standardized data collection form. Variables included age, sex, underlying conditions (e.g., intestinal failure, malignancy, transplantation), admitting service, length of hospital stay, pediatric intensive care unit (PICU) admission, and exposure to invasive procedures.

Data on CVC presence and removal, total parenteral nutrition, recent surgery, antimicrobial exposure, chemotherapy, immunosuppressive therapy, and antifungal prophylaxis were recorded. Laboratory parameters at candidemia onset included complete blood count indices, renal and liver function tests, inflammatory markers, and serum electrolytes.

The timing of CVC removal was documented and analyzed as a predefined management variable in relation to crude 30-day mortality. Among CLABSI episodes, CVC removal timing was additionally stratified using predefined time thresholds (≤48 h and ≤72 h from the date of candidemia diagnosis) for subgroup analyses. Elevated liver function tests were defined as serum aspartate aminotransferase (AST) and/or alanine aminotransferase (ALT) levels exceeding the age-adjusted upper limit of normal at the time of candidemia diagnosis. Elevated renal function tests were defined as serum urea and/or creatinine levels exceeding the age-adjusted upper limit of normal.

### 2.4. Microbiological Methods

Blood cultures were processed using the BACT/ALERT system (bioMérieux, Marcy-l’Étoile, France). Species identification was performed using conventional phenotypic methods and the ID32C (bioMérieux, Marcy-l’Étoile, France) system until 2014, and matrix-assisted laser desorption/ionization time-of-flight (MALDI-TOF) mass spectrometry thereafter. Antifungal susceptibility testing was conducted using the broth microdilution method according to Clinical and Laboratory Standards Institute (CLSI) criteria applicable during the study period.

### 2.5. Statistical Analysis

Continuous variables were summarized as medians with interquartile ranges (IQRs), and categorical variables as counts and percentages. Comparisons between survivors and non-survivors were performed using the Mann–Whitney U test for continuous variables and the chi-square or Fisher’s exact test for categorical variables, as appropriate.

Variables associated with crude 30-day mortality in univariable analyses (*p* < 0.10), together with clinically relevant covariates determined a priori, were entered into a multivariable logistic regression model to identify factors independently associated with mortality. Multicollinearity was assessed prior to model construction. To enhance interpretability, platelet count was modeled per 10,000/µL increase. Multivariable analyses were conducted as complete-case analyses; episodes with missing data for any included covariate were excluded from the regression model. Adjusted odds ratios (aORs) with 95% confidence intervals (CIs) were reported.

To address the time-dependent nature of catheter removal, a sensitivity Cox regression analysis was performed among episodes classified as CLABSI. In this model, CVC removal was entered as a time-dependent covariate, with episodes considered unexposed until the day of catheter removal and exposed thereafter.

Kaplan–Meier survival curves were constructed to evaluate 30-day survival according to CVC removal status and selected clinical variables, and differences between groups were assessed using the log-rank test.

Temporal trends in the proportions of non-*albicans Candida*, *Candida parapsilosis*, and *Candida albicans* were evaluated using binary logistic regression, with calendar year entered as a continuous variable. Separate models were fitted for each species or group versus the corresponding comparator group, and odds ratios were reported per one-year increase.

All statistical analyses were performed using IBM SPSS Statistics version 25 (IBM Corp., Armonk, NY, USA). A two-sided *p* value < 0.05 was considered statistically significant.

### 2.6. Ethics

The study was approved by the Ethics Committee of Ege University Faculty of Medicine (approval number: 26-1T/76). Given the retrospective design and use of anonymized routinely collected clinical data, the requirement for written informed consent from patients and/or their parents or legal guardians was waived by the ethics committee in accordance with national regulations and the principles of the Declaration of Helsinki. No identifiable patient information is reported in this manuscript.

## 3. Results

### 3.1. Baseline Characteristics

During the 18-year study period, 465 pediatric candidemia episodes were identified in 395 unique pediatric patients. Most patients had a single included episode (352/395, 89.1%), whereas 43 patients (10.9%) had more than one included episode. Baseline demographic, clinical, and management characteristics are summarized in Table 1. Candidemia was most frequently observed in Pediatric Surgery, Gastroenterology, and the PICU.

A CVC was present in 411 episodes (88.4%) at the time of diagnosis, and catheter removal was performed in 264 episodes (56.8%). Among catheterized episodes, 341 fulfilled criteria for CLABSI and were included in catheter-removal timing analyses; the remaining catheterized episodes did not meet CLABSI criteria because Candida growth was detected only in peripheral blood cultures without concomitant catheter blood culture positivity. Crude 30-day all-cause mortality was 10.8%.

**Figure 1:**
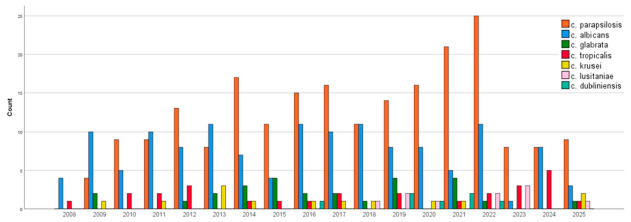
Annual distribution of *Candida* species isolated from pediatric candidemia episodes over the 18-year study period.

NAC species predominated, with *Candida parapsilosis* (46%) and *Candida albicans* (29%) representing the most frequent isolates. Temporal trends in species distribution across the study period are shown in Figure 1. In trend analyses using calendar year as a continuous variable, the overall proportion of non-*albicans Candida* increased significantly over the study period (OR per year, 1.079; 95% CI, 1.032–1.128; *p* = 0.001). The proportion of *Candida parapsilosis* did not change significantly over time (OR per year, 1.041; 95% CI, 1.000–1.084; *p* = 0.051), whereas the proportion of *Candida albicans* decreased significantly over time (OR per year, 0.927; 95% CI, 0.886–0.969; *p* = 0.001).

### 3.2. Comparison of Survivors and Non-Survivors by 30-Day Crude Mortality

Comparisons according to 30-day crude mortality are presented in Table 2 and Table 3. No significant differences were observed between survivors and non-survivors with respect to age, sex, or duration of hospitalization prior to candidemia onset. Non-survivors more frequently required intensive care and invasive supportive care and had higher rates of selected host vulnerability markers, including *Candida* colonization, chemotherapy, and immunosuppressive therapy.

Catheter removal was performed significantly less frequently in non-survivors compared with survivors (29.5% vs. 68.8%, *p* < 0.001). In analyses restricted to episodes classified as CLABSI according to NHSN criteria (*n* = 341), crude 30-day mortality differed significantly according to catheter management strategy. Mortality was 13.1% (31/237) among patients without early catheter removal and 4.8% (5/104) among those whose catheter was removed within 72 h (*p* = 0.022). When applying a 48 h threshold, mortality was 12.3% (34/276) among patients without catheter removal within 48 h and 3.1% (2/65) among those whose catheter was removed within 48 h (*p* = 0.029). In a sensitivity Cox regression analysis restricted to CLABSI episodes (*n* = 341), CVC removal modeled as a time-dependent covariate remained associated with lower 30-day mortality hazard (HR, 0.40; 95% CI, 0.18–0.86; *p* = 0.018).

At candidemia onset, non-survivors had more frequent severe neutropenia and thrombocytopenia, lower platelet counts, and higher markers of renal dysfunction and systemic physiological derangement. Detailed laboratory comparisons are shown in Table 3.

Antifungal susceptibility testing was performed in 363 isolates. No significant association was observed between 30-day mortality and susceptibility distributions for fluconazole, amphotericin B, caspofungin, or anidulafungin. Detailed susceptibility distributions are presented in Supplementary Table S1.

Species-specific crude 30-day mortality rates are presented in Table 4. Although crude mortality appeared lower for *C. parapsilosis*, the most common species in the cohort, and higher in some less frequent species groups, these comparisons were based on small numbers in several categories and should be interpreted descriptively rather than as evidence of independent species-specific mortality effects.

### 3.3. Kaplan–Meier Survival Analyses

**Figure 2:**
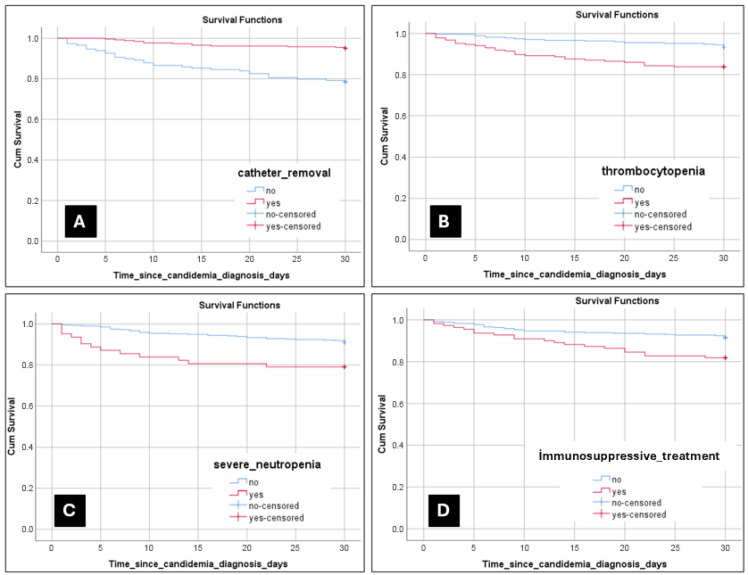
Kaplan–Meier estimates of 30-day survival following pediatric candidemia. Kaplan–Meier survival curves according to (**A**) central venous catheter removal, (**B**) thrombocytopenia (platelet count < 150 × 10^3^/µL), (**C**) severe neutropenia (absolute neutrophil count < 0.5 × 10^3^/µL), and (**D**) receipt of immunosuppressive therapy. Time was calculated from the date of candidemia diagnosis to death; patients alive at 30 days were censored. Survival distributions were compared using the log-rank test: (**A**) *p* < 0.001, (**B**) *p* = 0.001, (**C**) *p* = 0.002, and (**D**) *p* = 0.004.

Kaplan–Meier survival curves demonstrated significant separation in 30-day survival according to CVC removal status (Figure 2). Patients without catheter removal had significantly lower survival compared with those who underwent removal (log-rank *p* < 0.001).

Similarly, thrombocytopenia (platelet count < 150 × 10^3^/µL) was associated with reduced 30-day survival. Severe neutropenia (ANC < 0.5 × 10^3^/µL) was also associated with significantly lower survival probabilities. Receipt of immunosuppressive therapy was likewise associated with reduced survival.

### 3.4. Multivariable Analysis

In multivariable logistic regression analysis (complete-case analysis; *n* = 385), failure to remove the CVC remained the strongest independent factor associated with crude 30-day mortality (adjusted odds ratio [aOR] 6.63, 95% CI 2.85–15.42; *p* < 0.001) (Table 5).

In addition, higher serum urea levels and higher serum sodium levels were independently associated with mortality. Higher platelet counts were independently associated with lower mortality risk.

## 4. Discussion

In this 18-year retrospective cohort including 465 pediatric candidemia episodes, we provide a long-term single-center evaluation integrating epidemiology, management practices, and independent predictors of 30-day crude mortality. Despite a high burden of underlying disease and frequent exposure to invasive supportive care, crude 30-day mortality was 10.8%, falling toward the lower range reported in pediatric cohorts [6,9,15,16,17]. Overall, mortality in our cohort was more strongly associated with host vulnerability and modifiable management factors than with microbiological characteristics alone.

### 4.1. Source Control and Catheter Management

Among all evaluated variables, catheter management emerged as the most clinically actionable determinant of outcome. In the overall cohort, catheter removal was significantly less frequent among non-survivors than survivors (29.5% vs. 68.8%), and failure to remove the catheter remained independently associated with a more than six-fold increased risk of death after multivariable adjustment. Kaplan–Meier analyses further demonstrated significantly reduced 30-day survival in patients whose catheter was retained.

In analyses restricted to the 341 episodes classified as central line-associated bloodstream infections according to NHSN criteria, early catheter removal was associated with markedly lower crude 30-day mortality. Mortality was 13.1% among patients without removal within 72 h compared with 4.8% among those whose catheter was removed within 72 h. When applying a 48 h threshold, mortality was 12.3% without removal within 48 h versus 3.1% with removal within 48 h. These subgroup findings indicate that, among episodes fulfilling central line-associated bloodstream infection criteria, catheter retention or delayed removal consistently identified the highest-risk group.

These findings are concordant with prior studies demonstrating worse outcomes when catheter removal is delayed beyond the early treatment period [5,18,19,20,21], and align with current international guideline recommendations emphasizing prompt source control in candidemia management [11,12,22]. The observed association is biologically plausible, as intravascular devices provide a substrate for Candida biofilm formation, promoting persistent bloodstream infection and reduced antifungal efficacy [18,19].

Because catheter removal and its timing represent time-dependent exposures, patients must survive long enough for catheter removal to be performed. Therefore, children who died very early may have had less opportunity for catheter removal, and residual immortal time bias may influence the magnitude of observed associations. To address this issue, we performed a sensitivity Cox regression analysis among CLABSI episodes, modeling CVC removal as a time-dependent covariate. In this analysis, CVC removal remained associated with lower 30-day mortality hazard. Accordingly, although residual confounding cannot be excluded and the findings should still be interpreted as associative rather than causal, the consistency of results across 48 h and 72 h thresholds, CLABSI-restricted analyses, survival curves, multivariable modeling, and time-dependent Cox analysis supports the central clinical importance of timely source control in pediatric candidemia.

### 4.2. Host Vulnerability and Physiological Derangement

Mortality was closely linked to host complexity and markers of acute physiological compromise. Non-survivors exhibited higher rates of immunosuppressive therapy and invasive supportive care, consistent with prior pediatric studies identifying intensive care exposure and immunosuppression as correlates of mortality [17,23,24]. Severe neutropenia and thrombocytopenia were more frequent among non-survivors, and higher serum urea levels together with lower platelet counts remained independently associated with mortality in multivariable analysis. These findings are consistent with broader candidemia literature demonstrating that renal dysfunction, hematologic abnormalities, and critical illness reflect systemic disease severity rather than pathogen-specific virulence [6,11,25].

### 4.3. Species Distribution and Microbiological Factors

Non-albicans *Candida* species predominated throughout the study period, with *Candida parapsilosis* accounting for the largest proportion of bloodstream isolates, in line with pediatric epidemiological patterns reported globally [1,9,10,11]. Although crude mortality varied across species, these comparisons should be interpreted descriptively only, because several species groups included few episodes and the analyses were not designed to establish independent species-specific mortality effects. Trend analyses showed a significant increase in non-*albicans Candida* and a significant decrease in *C. albicans* over time, whereas *C. parapsilosis* did not show a statistically significant temporal change.

The highest crude mortality was numerically observed among episodes due to *Candida krusei* (3/13, 23.1%); however, the small number of cases limits definitive conclusions. In this cohort, species distribution appeared to reflect healthcare exposure and underlying host characteristics, while short-term mortality was more strongly associated with host vulnerability and catheter management than with species identity alone [26].

### 4.4. Antifungal Resistance

Antifungal susceptibility patterns were not consistently associated with crude 30-day mortality in analyses restricted to isolates with available susceptibility results for the corresponding antifungal agent. Similar dissociation between in vitro susceptibility and early clinical outcome has been described in pediatric and mixed-age studies, where mortality is more strongly driven by host vulnerability, illness severity, and adequacy of source control [1,6,11,27]. National pediatric data from Turkey likewise suggest that resistance does not consistently translate into higher early mortality when appropriate therapy and effective source control are achieved [9,24,28].

Initial antifungal treatment patterns differed between survivors and non-survivors; however, this finding should be interpreted cautiously. Because the cohort spans 2008–2025, antifungal management should also be interpreted in the context of evolving antifungal availability, local prescribing practices, guideline recommendations, and susceptibility testing methodologies. Seven non-survivors died before systemic antifungal therapy could be initiated, and treatment choice was likely influenced by illness severity, underlying conditions, local practice patterns, and changes in antifungal availability over the long study period. Therefore, our data do not allow causal inference about the comparative effectiveness of specific antifungal regimens.

### 4.5. Clinical Implications

Taken together, our findings indicate that pediatric candidemia represents a heterogeneous clinical entity in which early mortality is predominantly determined by host vulnerability and modifiable management strategies. Although this study was not designed to predict the occurrence of candidemia in children without bloodstream infection, several readily available clinical and laboratory parameters may support early risk stratification after candidemia is diagnosed. Immunosuppressive therapy, intensive care exposure, invasive supportive care, severe neutropenia, thrombocytopenia, elevated serum urea, and catheter retention or delayed source control should alert clinicians to a higher risk of early mortality. These factors may help guide closer monitoring, reassessment of antifungal adequacy, and early multidisciplinary evaluation for source control. However, these findings should not be interpreted as evidence for the superiority of any specific antifungal regimen. Our findings should be regarded as hypothesis-generating and require prospective external validation before use as a formal early warning system. These results support prioritizing early risk stratification, prompt initiation of appropriate antifungal therapy, and guideline-concordant catheter management in pediatric candidemia care [11,12].

### 4.6. Strengths and Limitations

Key strengths of this study include the large cohort size, the extended 18-year observation period, and integration of detailed clinical management variables with microbiological data. The combined use of multivariable logistic regression, Kaplan–Meier survival analyses, CLABSI-restricted timing analyses, and sensitivity Cox regression with catheter removal modeled as a time-dependent covariate enhances internal consistency and provides additional clinical insight.

Several limitations merit consideration. The retrospective single-center design introduces the possibility of residual confounding and limits generalizability. Evolving antifungal prescribing practices, antifungal availability, guideline recommendations, susceptibility breakpoints, and laboratory testing methodologies over the long study period may have influenced management patterns, susceptibility-related analyses, and outcomes. In addition, the retrospective dataset did not systematically capture all time-varying parameters reflecting progress in candidemia care, such as antifungal stewardship practices, catheter-care protocols, diagnostic work-up, supportive care practices, and changes in patient case mix; temporal changes in outcomes should be interpreted cautiously and should not be considered direct evidence of improvement in candidemia care. Quantitative blood cultures, catheter-tip cultures, and differential time-to-positivity analyses were not systematically performed; therefore, microbiological confirmation of catheter source beyond surveillance-based central line-associated bloodstream infection classification was not possible. Although we performed a sensitivity Cox regression analysis modeling CVC removal as a time-dependent covariate, residual immortal time bias and confounding by indication cannot be fully excluded because of the retrospective observational design. Multivariable analyses were conducted as complete-case analyses, and some selection bias related to missing data is possible. Finally, in critically ill children with malignancy or multiple comorbidities, mortality is often multifactorial, making precise attribution to candidemia challenging; for this reason, we selected 30-day crude all-cause mortality as the primary endpoint, consistent with prior pediatric invasive candidiasis studies [13].

## 5. Conclusions

In this long-term pediatric cohort, short-term mortality was primarily associated with host vulnerability and modifiable management factors rather than microbiological characteristics alone. Timely and effective source control, particularly CVC removal when feasible, emerged as the strongest modifiable factor associated with early survival. These findings support prioritizing early risk stratification and guideline-concordant catheter management in the care of children with candidemia.

## Figures and Tables

**Table 1:** Demographic, clinical, and management characteristics of pediatric candidemia episodes.

Demographic and Clinical Characteristics	*n* = 465, *n* (%)
Age, years, median (IQR)	2.10 (0.64–7.23)
Gender, male	275 (59.1)
Pre-candidemia hospital stay, days, median (IQR)	23 (10–46.75)
Post-candidemia hospital stay, days, median (IQR)	33 (18–60)
Total hospital stay, days, median (IQR)	63 (36–111.75)
PICU transfer due to candidemia episode	35 (7.5)
**Source of positive blood culture**	
Peripheral blood only	124 (26.7)
Catheter blood only	158 (34)
Both peripheral and catheter	183 (39.4)
**Department**	
Pediatric Surgery	108 (23.2)
Gastroenterology	101 (21.7)
PICU	89 (19.1)
Oncology	50 (10.8)
Hematology	24 (5.2)
Bone Marrow Transplantation Center	24 (5.2)
Others	69 (14.8)
**Underlying medical conditions**	
Intestinal failure	114 (24.5)
Bone marrow or solid organ transplantation	53 (11.4)
Solid malignancy	40 (8.6)
Hematologic malignancy	37 (8.0)
Others	221 (47.5)
**Clinical procedures and other risk factors**	
Central venous catheter	411 (88.4)
Catheter lock therapy	23 (4.9)
Catheter removal	264 (56.8)
Total parenteral nutrition	240 (51.6)
Gastric surgery	195 (41.9)
Candida colonisation	51 (11.0)
Antifungal prophylaxis	118 (25.4)
Breakthrough candidemia	162 (34.8)
**Outcome**	
30-day crude mortality	50 (10.8)
14-day attributable mortality	36 (7.7)

Abbreviations: IQR, interquartile range; PICU, pediatric intensive care unit.

**Table 2:** Baseline Characteristics and Clinical Management According to 30-Day Crude Mortality.

Demographic and Clinical Characteristics	Survivors (*n* = 415), *n* (%)	Non-Survivors (*n* = 50), *n* (%)	*p*-Value	OR (95% CI)
Age, years, median (IQR)	2.1 (0.65–7.02)	1.68 (0.65–8.15)	0.861	
Gender, male	243 (58.6)	32 (64)	0.459	
Pre-candidemia hospital stay, days, median (IQR)	24.0 (10.0–46.0)	17.5 (8.0–60.5)	0.757	
Post-candidemia hospital stay, days, median (IQR)	37.0 (20.8–69.0)	9.0 (5.0–19.3)	**<0.001**	
Total hospital stay, days, median (IQR)	66.5 (40.0–114.0)	35.0 (19.3–80.0)	**<0.001**	
PICU stay	270 (65.1)	39 (78.0)	**0.044**	1.904 (0.947–3.830)
PICU length of stay, days, median (IQR)	19.0 (7.0–44.0)	10.0 (4.0–24.0)	0.059	
Persistent candidemia episode	64 (15.4)	5 (10)	0.308	
Time to microbiological clearance, days, median (IQR)	6 (3–11)	4.5 (2.75–7)	0.064	
**Clinical procedures and risk factors**				
Central venous catheter	366 (88.2)	45 (90)	0.706	
Catheter removal	251 (68.8)	13 (29.5)	**<0.001**	0.190 (0.096–0.378)
Time to catheter removal, days, median (IQR)	4 (2–8)	4 (3–8)	0.942	
Urinary catheter	128 (30.8)	26 (52)	**0.003**	2.429 (1.343–4.393)
Mechanical ventilation	100 (24.1)	21 (42)	**0.006**	2.281 (1.246–4.177)
Total parenteral nutrition	214 (51.6)	26 (52)	0.954	
Gastric surgery	177 (42.7)	18 (36)	0.368	
Candida colonisation	36 (8.7)	9 (18)	**0.035**	2.311 (1.040–5.135)
Chemotherapy	78 (18.8)	16 (32)	**0.028**	2.033 (1.069–3.868)
Immunosuppressive therapy	90 (21.7)	20 (40)	**0.004**	2.407 (1.305–4.40)
Breakthrough candidemia	141 (34)	21 (42)	0.261	
**Initial antifungal therapy**				
Fluconazole	178 (42.9)	16 (32)	**<0.001**	
Echinocandin	125 (30.1)	17 (34)		
Amphotericin B	104 (25.1)	9 (18)		
Voriconazole	8 (1.9)	1 (2)		
None *	0	7 (14)		
Antifungal monotherapy †	374 (90.1)	37 (86.0)	0.425	
Combination antifungal therapy †	41 (9.9)	6 (14.0)	0.425	
Total duration of antifungal therapy, days, median (IQR)	17 (14–23)	10.5 (5–17.25)	**<0.001**	

* ‘None’ refers to patients who died before the initiation of systemic antifungal treatment. † Analysis of antifungal treatment strategy was restricted to episodes in which systemic antifungal therapy was initiated; seven untreated episodes were excluded. The *p* value refers to the comparison between monotherapy and combination therapy and was calculated using Fisher’s exact test. Bold values indicate statistical significance at *p* < 0.05.

**Table 3:** Laboratory Parameters at Candidemia Onset According to 30-Day Crude Mortality.

Laboratory Findings	Survivors (*n* = 415), *n* (%)	Non-Survivors (*n* = 50), *n* (%)	*p*-Value	OR (95% CI)
WBC (/µL), median (IQR)	7930 (4050–12690)	7350 (1070–16100)	0.728	
ANC (/µL), median (IQR)	4315 (1907–8160)	3810 (310–12000)	0.626	
Platelet count (/µL), median (IQR)	219,000 (88,250–329,250)	103,000 (32,750–183,000)	**<0.001**	
CRP (mg/L), median (IQR)	5.5 (1.4–13.5)	7.35 (2.42–17.97)	0.124	
Procalcitonin (ng/mL), median (IQR)	0.59 (0.21–2.17)	2.3 (0.69–12.5)	**0.044**	
AST (U/L)	39 (24–59.2)	73 (25.25–138)	**0.003**	
ALT (U/L)	29 (16–50)	51 (18–99)	**0.022**	
Urea (mg/dL)	23 (14.25–34)	48.5 (25–82)	**<0.001**	
Creatinine (mg/dL)	0.27 (0.19–0.4)	0.4 (0.2–0.82)	**0.003**	
Sodium (mmol/L)	137 (134–140)	140 (134–146.5)	**0.002**	
Neutropenia (ANC < 1.5 × 10^3^/µL)	87 (21.4)	15 (31.3)	0.123	
Mild neutropenia (ANC 1.0–1.5 × 10^3^/µL)	17 (4.2)	0	0.237	
Moderate neutropenia (ANC 0.5–1.0 × 10^3^/µL)	21 (5.2)	2 (4.2)	0.764	
Severe neutropenia (ANC < 0.5 × 10^3^/µL)	49 (12.1)	13 (27.1)	**0.004**	2.706 (1.340–5.467)
Duration of neutropenia, days, median (IQR)	8 (4–19.25)	8 (4–20)	0.830	
Thrombocytopenia, (<150 × 10^3^/µL)	155 (38.2)	30 (62.5)	**<0.001**	2.699 (1.455–5.006)
Elevated renal function tests	13 (3.1)	6 (12)	**0.003**	4.206 (1.523–11.62)
Elevated liver function tests	39 (9.4)	9 (18)	**0.060**	2.111 (0.955–4.666)

Abbreviations used in Table 2 and Table 3: OR, odds ratio; IQR, interquartile range; PICU, pediatric intensive care unit; WBC, white blood cell count; ANC, absolute neutrophil count; CRP, C-reactive protein; AST, aspartate aminotransferase; ALT, alanine aminotransferase. Bold values indicate statistical significance at *p* < 0.05.

**Table 4:** Species-specific crude mortality rates.

Candida Species	*n* (%)	Deaths, *n*	Crude 30-Day Mortality (%)
*C. parapsilosis*	214 (46.0)	17	7.9
*C. albicans*	135 (29.0)	15	11.1
*C. glabrata*	27 (5.8)	4	14.8
*C. tropicalis*	27 (5.8)	4	14.8
*C. krusei*	13 (2.8)	3	23.1
*C. kefyr*	13 (2.8)	0	0
*C. lusitaniae*	10 (2.2)	1	10
*C. dubliniensis*	7 (1.5)	1	14.3
*C. guilliermondii*	4 (0.9)	0	0
Unspecified	6 (1.3)	5	83.3
Other/rare species	9 (1.9)	0	0

Data are presented as *n* (%) for distribution and species-specific crude 30-day mortality rates. Given the limited number of episodes in several species categories, findings should be interpreted descriptively.

**Table 5:** Multivariable logistic regression analysis of factors independently associated with 30-day crude mortality in pediatric candidemia (complete-case analysis; *n* = 385).

Variable	aOR	95% CI	*p* Value
Failure to remove central venous catheter	6.63	2.85–15.42	<0.001
Serum urea (per 1 mg/dL increase)	1.03	1.02–1.04	<0.001
Serum sodium (per 1 mmol/L increase)	1.10	1.03–1.18	0.003
Platelet count (per 10,000/µL increase)	0.96	0.93–0.99	0.011

Abbreviations: aOR, adjusted odds ratio; CI, confidence interval. Variables were selected a priori based on clinical relevance and univariable analysis (*p* < 0.10).

## Data Availability

The data that support the findings of this study are not publicly available due to privacy and ethical restrictions. Data may be made available from the corresponding author upon reasonable request and with appropriate institutional approval.

## Supplementary Materials

The following supporting information can be downloaded at: https://www.mdpi.com/article/10.3390/jof12060445/s1. Table S1: Antifungal susceptibility patterns according to 30-day mortality.
