# Supplementary material for: Clinical Characteristics, Management, and 30-Day Mortality Predictors in an 18-Year Pediatric Candidemia Cohort
Source: J Fungi (Basel). 2026 Jun 17;12(6):445. doi: 10.3390/jof12060445 (PMC13301751; doi:10.3390/jof12060445)
Supplement: Supplementary file 1 [file jof-12-00445-s001.zip › jof-4348097-supplementary.pdf]

Supplementary Table S1. Antifungal susceptibility patterns according to 30-day mortality.

**Antifungal susceptibility**	**Total (*n* = 465), n (%)**	**Survivors (*n* = 415), n (%)**	**Non-survivors (*n* = 50), *n* (%)**	**p value**
Not tested	102 (21.9)	88 (21.2)	14 (28.0)	0.273
Tested	363 (78.1)	327 (78.8)	36 (72.0)	
**Fluconazole susceptibility**	***n* = 331**	***n* = 297**	***n* = 34**	
Susceptible	212 (64.0)	192 (64.6)	20 (58.8)	0.627
Susceptible dose-dependent	15 (4.5)	14 (4.7)	1 (2.9)	
Resistant	104 (31.4)	91 (30.6)	13 (38.2)	
**Voriconazole susceptibility**	***n* = 324**	***n* =292**	***n* = 32**	
Susceptible	241 (74.4)	218 (74.7)	23 (71.9)	**0.018**
Susceptible dose-dependent	12 (3.7)	8 (2.7)	4 (12.5)	
Resistant	71 (21.9)	66 (22.6)	5 (15.6)	
**Posaconazole susceptibility**	***n* = 177**	***n* = 165**	***n* = 12**	
Susceptible	145 (81.9)	134 (81.2)	11 (91.7)	0.639
Susceptible dose-dependent	4 (2.3)	4 (2.4)	0 (0.0)	
Resistant	28 (15.8)	27 (16.4)	1 (8.3)	
**Itraconazole susceptibility**	***n* = 323**	***n* = 290**	***n* = 33**	
Susceptible	207 (64.1)	189 (65.2)	18 (54.5)	0.216
Susceptible dose-dependent	39 (12.1)	32 (11.0)	7 (21.2)	
Resistant	77 (23.8)	69 (23.8)	8 (24.2)	
**Caspofungin susceptibility**	***n* = 173**	***n* = 162**	***n* = 11**	
Susceptible	159 (91.9)	150 (92.6)	9 (81.8)	0.426
Susceptible dose-dependent	8 (4.6)	7 (4.3)	1 (9.1)	
Resistant	6 (3.5)	5 (3.1)	1 (9.1)	
**Anidulafungin susceptibility**	***n* = 248**	***n* = 228**	***n* = 20**	
Susceptible	210 (84.7)	194 (85.1)	16 (80.0)	0.815
Susceptible dose-dependent	11 (4.4)	10 (4.4)	1 (5.0)	
Resistant	27 (10.9)	24 (10.5)	3 (15.0)	
**Amphotericin B susceptibility**	***n* = 330**	***n* = 297**	***n* = 33**	
Susceptible	313 (94.8)	282 (94.9)	31 (93.9)	0.893
Susceptible dose-dependent	1 (0.3)	1 (0.3)	0 (0.0)	
Resistant	16 (4.8)	14 (4.7)	2 (6.1)	

Data are presented as *n* (%), with percentages calculated within 30-day mortality groups (survivors and non-survivors). Analyses were restricted to isolates with available susceptibility results for the corresponding antifungal agent; isolates without susceptibility testing for that agent were excluded from the respective analysis. Antifungal susceptibility categories include susceptible, susceptible dose-dependent, and resistant. P values were calculated using the Pearson chi-square test. Given the limited number of outcome events, small subgroup counts in some susceptibility categories, and multiple comparisons across antifungal agents, P values should be interpreted cautiously. Although a nominal statistical difference was observed for voriconazole susceptibility distribution, voriconazole resistance was not more frequent among non-survivors, and no consistent or clinically interpretable association between antifungal susceptibility patterns and crude 30-day mortality was identified. No adjustment for multiple comparisons was performed.
